# Pain threshold in selected trigger points of superficial muscles of the back in young adults

**DOI:** 10.7717/peerj.12780

**Published:** 2022-02-01

**Authors:** Anna Katarzyna Cygańska, Paweł Tomaszewski, Anna Cabak

**Affiliations:** 1 Faculty of Rehabilitation, Józef Piłsudski University of Physical Education, Warsaw, Poland; 2 Faculty of Physical Education, Józef Piłsudski University of Physical Education, Warsaw, Poland

**Keywords:** Algometry, Pressure pain threshold, Reliability, Health, Case-control study, Trigger points

## Abstract

**Background:**

Monitoring of pain threshold is the basis for verification of the effectiveness of therapy or assessment of the patient’s condition. This study aimed to determine the pain threshold of selected superficial muscles of the back taking into account trigger point activity in young and healthy males and females, with the evaluation of intrarater reliability of algometric measurements.

**Material and methods:**

The study examined 30 young adult participants (15 males and 15 females) aged 26.23 ± 3.21, and BMI of 23.80 ± 3.43. The Pain Test FPX Algometer (Wagner) was used for the study. Trigger points on the levator scapulae and trapezius muscles (superior and inferior portion) on both sides were examined. It was also verified whether the trigger points studied are active or inactive. Furthermore, an author’s survey questionnaire was used.

**Results:**

Within the trigger points of the right (*p* = 0.04) and left (*p* = 0.02) superior trapezius muscle and the left (*p* = 0.04) levator scapulae muscle, the pain threshold values were higher in the male group. There was a statistically significantly higher number of active trigger points in the female group compared to that in the male group (2.49 ± 1.51 *vs*. 1.07 ± 1.16, respectively), *p* = 0.01. For all muscles tested, mean pain threshold values were significantly higher for inactive trigger points. A greater number of active trigger points is associated with lower pain thresholds at these points (left: the superior trapezius, *r* = −0.597, the inferior trapezius, *r* = −0.609; the levator scapulae, *r* = −0.746; right: the superior trapezius, *r* = −0.610, the inferior trapezius, *r* = −0.604; the levator scapulae, *r* = −0.747). The evaluation of the intrarater reliability showed excellent agreement between the first and second measurements, ICC > 0.987 for all examined trigger points.

**Conclusions:**

(1) Women who reported pain more than once a week in the studied muscles showed a greater number of active trigger points. (2) A greater number of active trigger points in female is related to a lower pain threshold (which is associated with greater pain sensitivity) in female than in male. (3) A sample size of 30 people seems sufficient to detect variations in the pain threshold at active and inactive trigger points of selected back muscles, especially when the frequency of occurrence of both types of points is comparable.

## Introduction

The most important issue in the field of physiotherapy is the development of easy and economical methods for the diagnosis of musculoskeletal disorders. Algometry fulfills both of the two criteria mentioned and is useful in clinical practice. Pressure pain threshold (PPT), measured by algometer is defined as the point at which painless stimulation such as pressure turns into a painful sensation. Pressure algometry (PA) is a method that objectively describes PPT (Pelfort et al., 2015). It allows for the quantification of pain and its assessment and evaluation in the context of the therapeutic process. As a method of diagnosis of pain sensitivity in soft tissues, algometry has gained increasing popularity among researchers in recent years (Wytrążek et al., 2015; Aboodarda, Spence & Button, 2015). It is considered an objective measurement method despite its subjective component, which consists in the subject’s assessment of the pain threshold. Some studies have used algometry to evaluate the effects of various therapeutic interventions based on pre- and post-intervention measurements (Pelfort et al., 2015; Andrzejewski et al., 2010; Kisilewicz et al., 2018; Tabatabaiee et al., 2019; Cygańska, Truszczyńska-Baszak & Tomaszewski, 2020), measurements within different parts of the body (Ylinen et al., 2007; Montenegro et al., 2012; Marciniak, 2018), or various disease entities (Van Wilgen, Van der Noord & Zwerver, 2011; Fredberg et al., 2004; Więckiewicz et al., 2015; Sæbø et al., 2020; Tabatabaiee et al., 2019; Mutlu & Ozdincler, 2015). There are also papers in the literature confirming good intrarater reliability of algometric measurements (Farasyn, Meeusen & Nijs, 2008; Waller et al., 2015; Walsh, Kinsella & McEvoy, 2017), although there are still many gaps in the knowledge to be filled. In particular, no normative PPT value for particular body parts among healthy subjects with sex differentiation has been established.

Musculoskeletal pain is associated with the presence of muscle trigger points (TrP) (Ribeiro et al., 2018). They can have an active (active trigger points) or inactive form (inactive trigger points/latent trigger points). An active trigger point is felt to be painful even without irritation of its area. It is responsible for the symptoms reported by the patient. An inactive trigger point does not produce clinical symptoms until it is irritated or compressed. Only when touched can the patient feel that a TrP is present in the area (Simons & Dommerholt, 2006). The sensitivity of tissues may change depending on many factors, *i.e*. their tension, ambient temperature, disease state taking place within them, age, gender, but also the general condition of the body and hygiene of everyday life related to sleeping, resting, nutrition, hydration, regular physical activity (Andrzejewski et al., 2010), or overload of the musculoskeletal system (Cabak et al., 2016). To monitor pain thresholds in different disease entities or to assess patient status and treatment effects, it is necessary to establish normative values of pain threshold in the populations of people with and without musculoskeletal complaints (Park et al., 2011). In available literature there is lack of objective differentiation of active and latent trigger points, and their PPT value. The potential findings of the presented study could help to understand relationship of PPT value of active and latent trigger points value in healthy males and females. Due to the high frequency of back/neck pain and the presence of trigger points within these muscles, they are particularly important in determining their pain threshold value (Ribeiro et al., 2018; Lucas, Rich & Polus, 2008). The hypothesis is that pain threshold values of selected muscle trigger points will be comparable in males and females with higher pain threshold values for inactive TrPs. It was also assumed that occurrence of active and inactive points will be the same in studied subjects.

Therefore, this study aimed to determine the pain threshold of selected superficial muscles of the back taking into account trigger point activity in young and healthy subjects, with the evaluation of intrarater reliability of algometric measurements. The study also determined the relationship between trigger point activity and reported pain.

## Materials and Methods

### Research population

The study design was approved by the Senate Ethics Committee for Scientific Research (SKE 01-13/2020) and was conducted according to the standards set by the Declaration of Helsinki (Williams, 2008). Thirty subjects who constituted a convenience sample were examined (15 males and 15 females); they were recruited form physiotherapy students from a local university. The subjects were informed about the purpose and procedures of the study and gave written, informed consent to participate in the study. The characteristics of the study group are presented in Table 1.

**Table 1:** Characteristics of the study group (Mean, SD).

Variable	Women (*n* = 15)	Men (*n* = 15)
Age (years)	26.2 ± 3.7	26.3 ± 2.6
Body mass (kg)	65.1 ± 12.4	74.3 ± 10.3
Body height (cm)	167.0 ± 9.0	175.0 ± 6.0
BMI	23.3 ± 3.9	24.2 ± 2.8
Sitting for 5–6 h a day (% of respondents)	40%	67%
Sitting for more than 7 h a day (% of respondents)	60%	33%

The inclusion criteria for the study were: age between 20 and 35 years, work/sedentary activity of 5 h per day. The exclusion criteria included acute inflammatory conditions with an elevated body temperature, use of analgesics on the day of the examination and within the previous week, use of drugs/relaxants within 4 months before the examination, current (up to 3 weeks) acute musculoskeletal pain and current (up to 3 weeks) musculoskeletal injuries and trauma, history of trauma (fracture, sprain, dislocation) in the upper limbs, head and cervical//thoracic spine, neurological diseases (including polyneuropathies, peripheral nerve damage, sensory disorders), circulatory disorders, skin lesions and skin diseases, the periods of pregnancy and menstruation in women (Bajaj et al., 2001), and professional sports training (Pettersen, Aslaksen & Pettersen, 2020). The exclusionary criteria were administered in the initial talk before measurements and assessed by researcher.

### Survey questionnaire

The participants were asked to fill out the author’s survey questionnaire prior to algometric measurements. The questionnaire consisted of 11 closed-ended questions: eight questions related to physical activity (type of physical activity, fitness level, frequency and duration of physical activity), two questions related to occupation (type of work performed, time spent in sedentary position), and one question related to the frequency of musculoskeletal complaints. The questions were multiple-choice, with appropriate choice ranges or response options. The average time spent by a participant to complete the questionnaire was approximately 5 min.

### Algometric measurement procedure

Algometric measurements were performed using the Pain Test FPX Algometer (Wagner Instruments, Riverside, CT, USA). The pain threshold was defined in kg/cm^2^ and the pressure was performed at a constant speed. All measurements were recorded by the same researcher (physiotherapist), trained in applying constant speed (50 kPa/s) who had about 1 year of experience in algometry measurements. The pain threshold of selected muscle trigger points was measured, and the locations of the trigger points were determined based on the methodology developed by Nie et al. (2005), Fig. 1. After the trigger points were palpated, they were marked using a skin marker to ensure reproducibility of the test point measurement. The levator scapulae muscle (designated as No. 5; 2 cm above the superior angle of the scapula, at the shoulder-neck transition) and the superior (designated as No. 4; midway between the spinous process of the C7 cervical vertebra and the shoulder process, above the crest of the scapula) and the inferior portions of the trapezius muscle (designated as No. 11; midway between the spinous process of the Th6 thoracic vertebra and the medial border of the scapula) were examined on both sides of the body. It was also verified whether the trigger points studied are active or inactive. An active trigger point (“painful without pressing it”) was considered to be a point that was painful, with the patient indicating pain in that area or when gently touching the skin (an active trigger point may lead to radiating pain in a specific area). An inactive trigger point (“painful when pressed”), on the other hand, was defined as a point not causing local pain or radiating pain without stimulation, with the pain revealed only during applying pressure (Ribeiro et al., 2018; Simons & Dommerholt, 2006; Nie et al., 2005; Calvo-Lobo et al., 2018).

**Figure 1:**
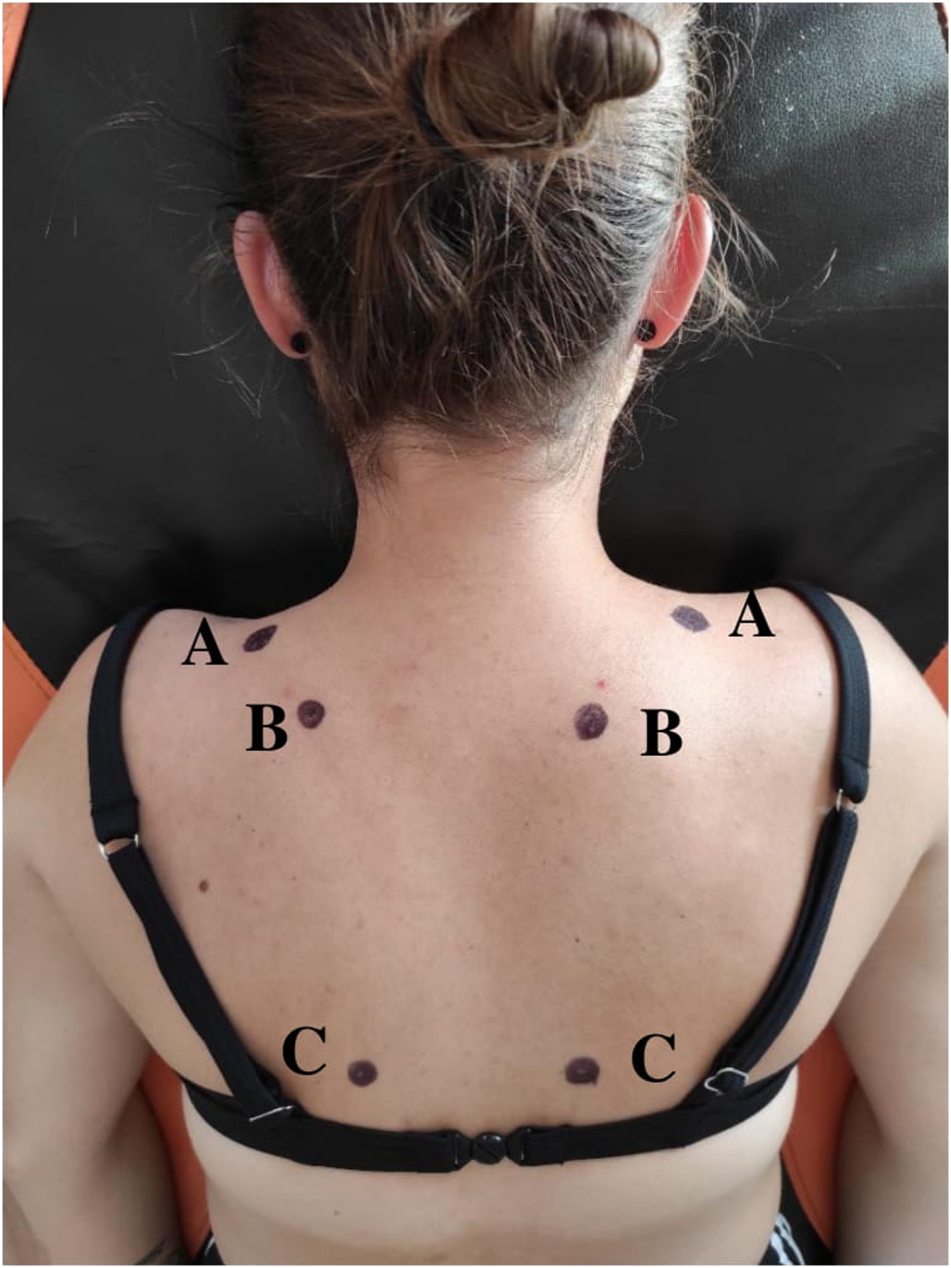
Location of trigger points on the right and left side of the neck and shoulder. Own source (A) Superior/descending portion of the trapezius muscle; (B) Levator scapulae muscle; (C) Inferior/ascending portion of the trapezius muscle.

Each measurement was conducted in the prone position (face placed in the opening of the bed, upper limbs along the trunk) by applying the device at the angle of 90° to the surface of the skin, always starting from the points on the right side. The subject was asked to say “STOP” when they felt the first distinct sensation of pain at the test point. Before measuring the actual points, a trial measurement was taken in the forearm muscles so that the subject could get an idea of the pain threshold sensation. The result was read by the same researcher after each measurement. The measurements were repeated 2 times at 5-min intervals. After algometric measurements, body weight and height were measured using an electronic balance with a scale.

### Statistical analysis

Basic statistical measures were used to describe the results: arithmetic means, standard deviations (SD), and percentages. The assumption of normality of the distributions of analyzed variables was verified by the Shapiro–Wilk test, whereas homogeneity of variance was verified by the Levene test. Because the assumptions of parametric tests were not met and the group sizes were relatively small, between-group differences were assessed using the Mann–Whitney test. The pain threshold values of selected muscle trigger points were dependent variables while sex and trigger point status (active/inactive) were independent variables. The *d*-equivalent measure (*d*_eq_) was calculated to express the effect size, as recommended for nonparametric procedures, small sample size and non-normal distribution (Rosenthal & Rubin, 2003). Additionally, a *post hoc* power analysis was performed. For categorical variables, the Fisher exact test was used. The relationships between the variables were tested using Spearman’s rank correlation. Correlation of pain threshold value with number of active trigger points in women and men was assessed, mean value of pain thresholds in assessed points was used in the analysis. Intraclass correlation (ICC) together with 95% confidence intervals (95% CI) was calculated to evaluate intrarater reliability. Analyses were performed using STATISTICA 13 (TIBCO Software, Palo Alto, CA, USA). The level of *p* < 0.05 was set to evaluate the significance of the effects.

## Results

### Survey questionnaire

In the study group (*n* = 30), 7 (23.3%) people had physical jobs, 9 (30%) people had intellectual jobs, and 14 (46.7%) people had mixed jobs. The time spent in the sitting position was between 5 and 8 h in 26 (86.6%) people, and more than 9 h in 4 (13.3%) people.

Only two subjects did not do any physical activity, and the remaining subjects declared undertaking regular physical activity (at least two times a week): 10 (33.3%) people were involved in cycling, seven (23.3%) people attended fitness classes, six (20%) people did roller-skating, and five (16.7%) people were involved in other activities. The subjects mostly rated their physical fitness as medium or high (24 people, accounting for 80%). Both intense and moderate exercise was undertaken by the subjects one to three times a week and its duration was similar, with 53.1 ± 33 *vs*. 67.3 ± 66.6 min, respectively. At least 10-min walks were taken two to three times a week by 10 (33.3%) people, and more than four times a week by 19 (63.3%) people.

Musculoskeletal complaints occurring several times a month (3–4 times) were reported by 12 subjects (40%), whereas in seven subjects (23%) pain occurred several times a week, almost every day. Only three people (10%) did not report any ailment and they were men (all women declared having ailments). Women more frequently reported pain compared to men; eight women reported pain several times a week or almost every day compared to only three men.

### Algometric measurement

There were statistically significant differences between women and men regarding the pain threshold in the area of the trigger point of the superior/descending portion of the trapezius muscle on the right and left sides (the differences being 1.82 and 2.09, respectively), and of the left and right levator scapulae muscle (5.19 ± 1.59 *vs*. 6.67 ± 1.99; *p* = 0.04) with the pain threshold values higher in the group of men. Detailed data are presented in Table 2.

**Table 2:** Pain threshold values (kg/cm^2^) of selected muscle trigger points in groups of women and men (arithmetic mean, SD).

Location	Women (*n* = 15)	Men (*n* = 15)	*U*	*p*-value	*d*_eq_
Right side					
Superior/descending portion of the trapezius	5.02 ± 1.06	6.84 ± 2.55	62.0	0.04	0.83
Inferior/ascending portion of the trapezius	7.40 ± 2.51	7.40 ± 3.28	105.5	0.77	0.11
Levator scapulae	5.32 ± 1.50	6.69 ± 1.16	70.0	0.08	0.68
Left side					
Superior/descending portion of the trapezius	5.02 ± 1.18	7.11 ± 2.22	57.0	0.02	0.93
Inferior/ascending portion of the trapezius	7.08 ± 2.54	7.95 ± 2.82	94.0	0.46	0.28
Levator scapulae	5.19 ± 1.59	6.67 ± 1.99	62.0	0.04	0.83

**Note:**

Differences tested by the Mann–Whitney *U* test; *U*, the Mann–Whitney test statistics; *d*_eq_, *d*-equivalent measure of effect size (Rosenthal & Rubin, 2003).

In the group of subjects (*n* = 30), the trigger points of the muscles studied were identified as active: one in 5 subjects (16.7%), two in 10 subjects (33.3%), three in 1 subject (3.3%), four in 5 subjects (16.7%), and five in 1 subject (3.3%). There was a statistically significantly higher (Mann–Whitney *U* = 52.0; *p* = 0.01; *d*_eq_ = 1.03) number of active trigger points in the female group compared to that in the male group (2.49 ± 1.51 *vs*. 1.07 ± 1.16, respectively). In the group of subjects in which a trigger point in the superior/descending portion of the left trapezius muscle was diagnosed as an active TrP (*n* = 10), the majority were female (80% *vs*. 20%); *p* = 0.05 by the Fisher exact test. Detailed data on the occurrence of active trigger points in the muscles studied is presented in Table 3. For all muscles tested, mean pain threshold values were significantly higher for inactive trigger points, the greatest differences were observed for inferior/ascending portion of the trapezius muscle (3.08 and 3.49 for right and left side respectively). The highest percentage of active trigger points was reported in the superior trapezius muscle (43%) and levator scapulae muscle (40%) on the right side. However, the greatest disproportion in the number of active and inactive points, with a significant advantage of the latter was found in the inferior trapezius muscle (right and left). Despite the relatively small group size, a satisfactory power of the test was obtained (>0.8) for most of the comparisons, whereas for the muscles in which significant disproportions in the occurrence of active and inactive points were found (inferior trapezius and left levator scapulae muscles), the power of the test estimated based on the results was slightly lower and ranged from 0.51 to 0.64.

**Table 3:** The occurrence of active trigger points in the muscles studied (%) and their pain threshold values (Mean, SD), *n* = 30.

Location	Active	Inactive	% of active	*U*	*p*-value	*d*_eq_
Right side						
Superior/descending portion of the trapezius	13 (4.48 ± 1.79)	17 (6.92 ± 1.84)	43%	31.0	0.00	1.53
Inferior/ascending portion of the trapezius	6 (5.31 ± 1.59)	24 (8.39 ± 2.84)	20%	25.5	0.01	0.98
Levator scapulae	12 (4.60 ± 1.08)	18 (6.94 ± 2.05)	40%	26.0	0.00	1.64
Left side						
Superior/descending portion of the trapezius	10 (4.18 ± 1.08)	20 (7.00 ± 1.74)	33%	14.0	0.00	1.91
Inferior/ascending portion of the trapezius	3 (4.38 ± 1.82)	27 (7.87 ± 2.54)	10%	8.0	0.02	0.90
Levator scapulae	9 (4.80 ± 1.21)	21 (6.41 ± 1.99)	30%	49	0.04	0.81

**Note:**

Differences tested by the Mann–Whitney *U* test; *U*, the Mann–Whitney test statistics; *d*_eq_, *d*-equivalent measure of effect size (Rosenthal & Rubin, 2003).

**Figure 2:**
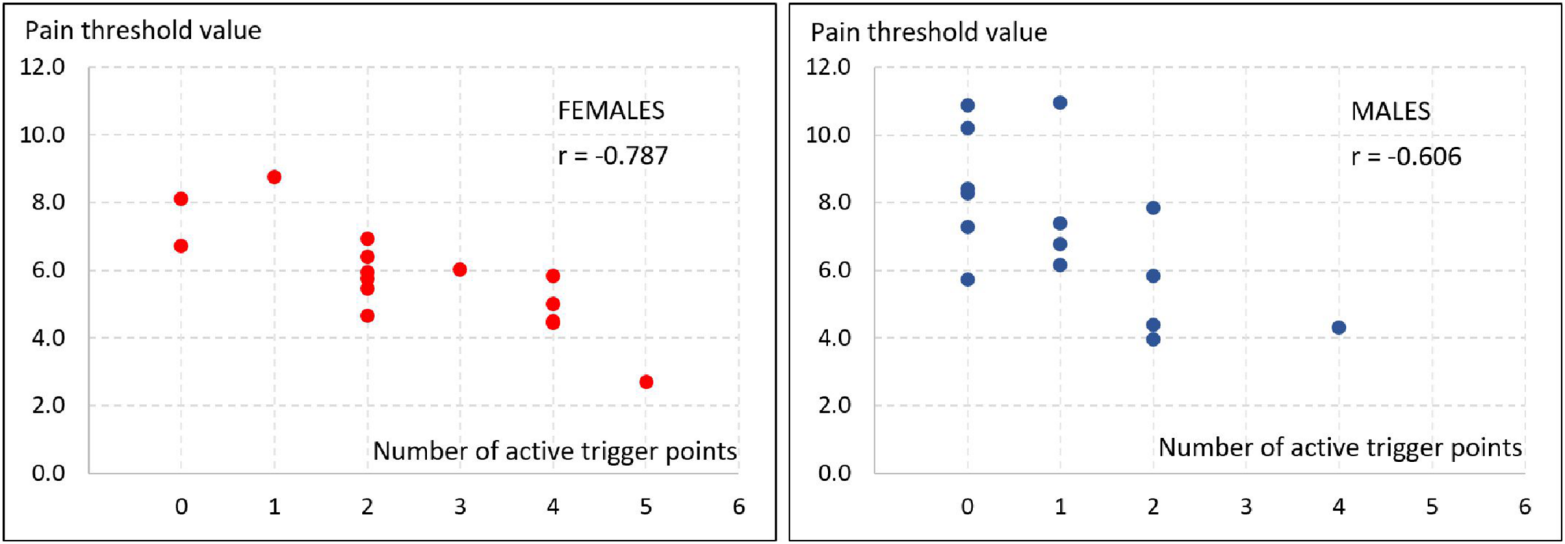
Relationships between the number of active trigger points and mean pain threshold values in female (*n* = 15) and male (*n* = 15) participants. *r* - Spearman’s rank correlation coefficient.

Significant correlations (*p* < 0.05) of the trigger points studied with the frequency of reported pain in the head, neck, and shoulder regions were found. The less frequently the pain was experienced, the higher the pain threshold values of the tested points were recorded in the measurements (left: superior trapezius, *r* = 0.396, *p* = 0.03; inferior trapezius, *r* = 0.431, *p* = 0.02; right: superior trapezius; *r* = 0.451, *p* = 0.01; inferior trapezius, *r* = 0.413, *p* = 0.02; levator scapulae, *r* = 0.413, *p* = 0.02). A greater number of active trigger points was also significantly (*p* < 0.001) associated with lower pain thresholds at these points (left: superior trapezius, *r* = −0.597, inferior trapezius, *r* = −0.609; levator scapulae, *r* = −0.746; right: superior trapezius, *r* = −0.610, inferior trapezius, *r* = −0.604; levator scapulae, *r* = −0.747). This was confirmed by significant correlations between the number of active trigger points and mean pain threshold values, showing a slightly higher coefficients in female (*r* = −0.787; *p* < 0.001) than in male (*r* = −0.606; *p* = 0.02) participants (Fig. 2). Furthermore, the less frequently pain was experienced by the participants, the lower the number of active trigger points (*r* = −0.608, *p* < 0.001).

The evaluation of the intrarater reliability showed high agreement between the first and second measurements, the ICC values ranged from 0.987 (95% CI [0.973–0.994]) to 0.993 (95% CI [0.986–0.997]) for ascending portion of the trapezius left side and descending portion of the trapezius right side, respectively. The reliability of the measurement is therefore at an excellent level (values greater than 0.90 indicate excellent reliability) (Koo & Li, 2016).

## Discussion

The analysis of the results concerning gender differences in pain thresholds reveals the need for a multi-faceted approach to these problems. Many researchers have confirmed that women have lower pain thresholds in the muscles studied but at the same time, many studies have failed to show this relationship (Chesterton et al., 2003). Some authors chose to study only women or only men (Ylinen et al., 2007; Lacourt, Houtveen & van Doornen, 2012). The present study demonstrated a significant difference in pain threshold between women and men for some muscle trigger points which is not entirely in line with the study hypothesis. The study also showed that pain threshold decreases with a higher number of active trigger points, whereas the fact that women had a statistically significantly higher number of active trigger points may suggest that the estimated lower pain threshold value in women may be due to these factors. In future studies, it will be worthwhile to take into account not only the sex but also the division into active and inactive points among men and women when assessing the mean value of the pain threshold. Women were also more likely to report experiencing pain compared to men, and this is associated with a lower pain threshold and a greater number of active trigger points. Therefore, in addition to gender, it would be appropriate to include the current level of pain as determined, for example, on the visual analogue scale (VAS) or the numeric rating scale (NRS), or to perform another additional measurement as a reference for algometric measurements (Lacourt, Houtveen & van Doornen, 2012; Alfonsin et al., 2019).

For all muscles tested, mean pain threshold values were significantly higher for inactive trigger points. This is in accordance with the hypothesis presented in the introduction. Similar results were obtained by Wytrążek et al. (2015) who confirmed lower pain threshold values if trigger points were present within the muscles tested. Furthermore, PPT results also correlated with an increase in amplitude during resting electromyography (rEMG). On the other hand, neither algometric measurements nor the use of resting electromyography (rEMG) and maximal contraction electromyography (mcEMG) allow for distinguishing the type of TrP. PPT results are also lower for pain in the sacroiliac joint (SIJ) (Van Leeuwen et al., 2016), in patients after conservative treatment of a wrist fracture (Sæbø et al., 2020), in female patients with endometriosis compared to patients with other causes of chronic pelvic pain (Alfonsin et al., 2019).

Lucas, Rich & Polus (2008) demonstrated that the average number of inactive trigger points is not related to gender, but is related to the side of the body (the dominant limb showed more trigger points), age (older people have more inactive trigger points), and muscle tested. Inactive trigger points were shown to be more prevalent in the serratus posterior muscle and the superior trapezius muscle compared to the rhomboid muscle, levator scapulae, pectoralis minor, or the inferior, and least frequently, the middle portions of the trapezius muscle. In our study, the highest percentage of inactive trigger points was found in the inferior trapezius muscle (right and left), moreover a significant disproportion in the number of active and inactive points was found. This is not in line with the adopted hypothesis that assumed equal occurrence of active and latent trigger points in studied subjects. Wytrążek et al. (2015) also demonstrated a more frequent occurrence of the nonreferring inactive/latent TrP in all of examined muscles (m. trapezius, m. sternocleidomastoid, m. infraspinatus, m. deltoideus) in young volunteers.

Regardless of the statistical methods used, several authors have confirmed good internal consistency, intrarater and interrater reliability of algometric measurements across body parts (Pelfort et al., 2015; Ylinen et al., 2007; Waller et al., 2015; Chesterton et al., 2003) or disease entities (Van Wilgen, Van der Noord & Zwerver, 2011; Więckiewicz et al., 2015; Mutlu & Ozdincler, 2015; Park et al., 2011). Our study also confirmed the excellent intrarater reliability. Furthermore, Lacourt, Houtveen & van Doornen (2012) demonstrated that the first measurement revealed significantly higher values compared to the second and third, while the value of the second and third measurements did not differ. They also showed that consistency is higher when we take bilateral points on the body part than the different points on the same side of the body.

The sample size for the algometric examinations was estimated by Waller et al. (2015) for different body regions (wrist, leg, neck, back) for crossover study design and parallel study design, and power at 80% and 90%. Neck (on the trapezius muscle, at the midpoint between the C7 spinous process and the lateral acromion) in his study corresponded to the location of the right (Waller et al., 2015 studied points on the right side only) trigger point of the superior trapezius muscle assessed in our study. The results obtained confirm that the sample size for the muscles of this body region is up to about 30 people, while the smaller the difference between the pain threshold in subsequent measurements, the higher the number of subjects. This in a way confirms our findings that for a large disproportion of active and inactive points within one muscle, *i.e*. with a significant difference in pain threshold, the number of subjects should be higher.

By describing the method of testing the pain threshold, authors provide the location of the structures tested (muscle trigger points, points of muscle attachment to bone, bone points, *etc*.) (Andrzejewski, Kassolik & Cymer, 2009; Fingleton et al., 2014; Frank, McLaughlin & Vaughan, 2013) and the method of palpation (flat palpation, pincer palpation, *etc*.) as well as the criteria of differentiation for the trigger points (good reliability for palpation of taut band, nodule, spot tenderness between 4 raters; very low reliability was found for the palpation of jump sign and the local twitch response (Lacourt, Houtveen & van Doornen, 2012); active, inactive, inactive radiating, inactive not radiating, *etc*.). Many researchers performed training and instruction in the use of an algometer prior to the actual measurement to obtain a specific rate of pressure and a trial measurement to allow the subject to get an idea of what the pain threshold is (Waller et al., 2015; Sciotti et al., 2001). Some authors take into consideration the maximum and minimum values of pressure, the number of times the measurement was repeated (and whether each measurement was included in the analysis, the average of all test, *etc*.; the first measurement has higher values compared to the second and third; Sæbø et al., 2020; Van Leeuwen et al., 2016), the intervals between the measurements, patient’s position during the measurement, the type and model of the algometer or the unit of measurement, and the number of researchers (sufficient reliability in palpation of TrP by two clinicians) (Sciotti et al., 2001), the gender of the researcher (Sæbø et al., 2020) and whether the researchers and subjects were blinded during the reading of the measurements (Sæbø et al., 2020; Sciotti et al., 2001). The multitude of factors and variables that potentially affect measurement results is a challenge. No studies have been found on the effect of measurement method on the measurement result, and there is no gold standard for measurement methodology. Review papers on trigger points that assessed publication quality indicated the need for research on large samples of subjects and strong study design (Ribeiro et al., 2018; Chesterton et al., 2003). This represents a space for continued research using algometry. The aim of the planned research will be to determine the pain threshold values of selected superficial muscles of the back on a large population of healthy young people. Limitations of the study. One limitation of the study is that some trigger points, after distinguishing into active and inactive, had small numbers where the differences in proportions were significant, *e.g*. 3:27 or 6:24. Additionally, the definition of active *vs*. inactive TrP presented in the literature is vague and in some cases overlapping as both trigger points appear to require some sort of mechanical stimulation to elicit a noxious response. Another limitation is the use of a relatively small sample size without *a priori* power analysis. The survey also has a subjective element: this can result in greater measurement variation, which is more disadvantageous with small sample numbers. In this study, the physical activity status was self-reported in the questionnaire survey. Perhaps the use of physical fitness tests could objectify the results obtained. The last technical limitation is the lack of a specific rate controller on the algometer, potentially adding inconsistent temporal delay upon the subjective designation of a PPT threshold. However, these limitations do not undermine the essence and value of the study. As a special value, it should be pointed out that based on the sample studied, the minimum numbers of subjects in groups divided into active and inactive trigger points were estimated. Furthermore, additional implications of a higher pain threshold in women have been demonstrated. The present research is continued with a larger sample size to establish normative values for the pain threshold for healthy adult women and men.

## Conclusions

1. Women who reported pain more than once a week in the studied muscles showed a greater number of active trigger points.
2. The present study demonstrated that grater amount of active trigger points in female is related to a lower pain threshold (which is associated with greater pain sensitivity) in female than in male.
3. A sample size of 30 people seems sufficient to detect variations in the pain threshold at active and inactive trigger points of selected back muscles, especially when the frequency of occurrence of both types of points is comparable. In future studies aimed to determine pain thresholds (cut-off points), a larger number of subjects should be examined, especially for the muscles where significant disparities in the occurrence of active and inactive trigger points may occur.

## Supplemental Information

10.7717/peerj.12780/supp-1 Supplemental Information 1 Raw data.

10.7717/peerj.12780/supp-2 Supplemental Information 2 Author’s questionnaire.

10.7717/peerj.12780/supp-3 Supplemental Information 3 English version of author’s questionaire.

## Additional Information and Declarations

### Data Availability

The following information was supplied regarding data availability: The raw data is available in the Supplemental Files.
