# Supplementary material for: Pain threshold in selected trigger points of superficial muscles of the back in young adults
Source: PeerJ. 2022 Feb 1;10:e12780. doi: 10.7717/peerj.12780 (PMC8815375; doi:10.7717/peerj.12780)
Supplement: Supplemental Information 2 [file peerj-10-12780-s002.pdf]

Załącznik 3.

KARTA BADANIA PACJENTA – karta własna

PYTANIA OGÓLNE

Imię i nazwisko ………………………………………………………

Płeć ………………………………………………………

Wiek ………………………………………………………

Wzrost ………………………………………………………

Waga ………………………………………………………

BMI (*body mass index)* ………………………………………………………

ANKIETA WŁASNA

1. Jaki rodzaj pracy wykonujesz?
   a. ☐Fizyczna
   b. ☐Umysłowa
   c. ☐Mieszana
2. Jaką aktywność fizyczną podejmujesz regularnie, tzn. <u>min. 2 razy w tygodniu</u>? (można wybrać kilka):
   a. ☐Siłownia
   b. ☐Bieganie
   c. ☐Rower
   d. ☐Fitness
   e. ☐Inne jakie? ……………………………………………………
3. Jak oceniasz swoją sprawność fizyczną?
   a. ☐Bardzo niska
   b. ☐Niska
   c. ☐Średnia
   d. ☒Wysoka
   e. ☐Bardzo wysoka

<u>Intensywny wysiłek fizyczny</u> wywołuje bardzo szybkie oddychanie i bardzo szybkie bicie serca – np. dźwiganie ciężkich przedmiotów, kopanie ziemi, aerobik, szybki bieg, szybka jazda rowerem. Co najmniej 10 min. bez przerwy.

4. Jak często wykonujesz czynności wymagające <u>intensywnego wysiłku fizycznego</u>?
   a. ☐Nigdy/nie jestem pewien(a)
   b. ☐1 raz w tygodniu lub rzadziej
   c. ☐2-3 razy w tygodniu
   d. ☐4 razy w tygodniu lub więcej
5. Przeciętnie ile czasu wykonujesz czynności wymagające <u>intensywnego wysiłku fizycznego</u> w ciągu takiego dnia?

a. ………….. minut dziennie
b. ☐Nie wiem/nie jestem pewien(a)

<u>Umiarkowany wysiłek fizyczny</u> prowadzi do trochę szybkiego oddychania i trochę szybkiego bicia serca – np. noszenie lżejszych ciężarów, jazda rowerem w normalnym tempie, gra w siatkówkę lub bardzo szybki marsz (nie chodzenie). Co najmniej 10 min. bez przerwy.

6. Jak często wykonujesz czynności wymagające <u>umiarkowanego wysiłku fizycznego</u>?
   a. ☐Nigdy/nie jestem pewien(a)
   b. ☐1 raz w tygodniu lub rzadziej
   c. ☐2-3 razy w tygodniu
   d. ☐4 razy w tygodniu lub więcej
7. Przeciętnie ile czasu wykonujesz czynności wymagające <u>umiarkowanego wysiłku fizycznego</u> w ciągu takiego dnia?
   a. ………….. minut dziennie
   b. ☐Nie wiem/nie jestem pewien(a)
8. Aktywności fizyczna, która jednorazowo wynosi <u>min. 45 min., tętno ulega podwyższeniu, powstaje zadyszka, odczuwalny jest pot na ciele</u>:
   a. ☐Nigdy/nie jestem pewien(a)
   b. ☐1 raz w tygodniu lub rzadziej
   c. ☐2-3 razy w tygodniu
   d. ☐4 razy w tygodniu lub więcej
9. Jak często <u>chodzisz co najmniej 10 min. bez przerwy</u>? *Interesuje nas chodzenie związane z pracą, chodzenie ulicą, np. zakupy, do pracy, a także o spacery.*
   a. ☐Nigdy/nie jestem pewien(a)
   b. ☐1 raz w tygodniu lub rzadziej
   c. ☐2-3 razy w tygodniu
   d. ☐4 razy w tygodniu lub więcej
10. Ile czasu dziennie spędzasz <u>siedząc</u>, biorąc pod uwagę dni powszednie? *Interesuje nas siedzenie przy biurku, siedzenie podczas odwiedzin u znajomych, podczas czytania, a także siedzenie lub leżenie podczas oglądania telewizji. Proszę uwzględnić czas spędzony na siedzeniu w domu, w pracy, w szkole, w pojazdach i w innych miejscach.*
   a. ☐4 h i mniej
   b. ☐5-6 h
   c. ☐7-8 h
   d. ☐9 h i więcej
11. Jak często odczuwasz dokuczliwe przeciążenia i dolegliwości bólowe okolic kręgosłupa, szyi, głowy, ramion (które ustępują same, ale są nieprzyjemne i utrzymują się dłużej niż jeden dzień)
   a. ☐Kilka razy w tygodniu, prawie codziennie
   b. ☐1-2 razy w tygodniu
   c. ☐Kilka razy w miesiącu (3-4)
   d. ☐Bardzo rzadko, nie w każdym miesiącu
   e. ☐Nie odczuwam wcale
