# Supplementary material for: Pain threshold in selected trigger points of superficial muscles of the back in young adults
Source: PeerJ. 2022 Feb 1;10:e12780. doi: 10.7717/peerj.12780 (PMC8815375; doi:10.7717/peerj.12780)
Supplement: Supplemental Information 3 [file peerj-10-12780-s003.docx]

**GENERAL QUESTIONS**

First name……………………………

Last name ……………………………

Sex……………………………………………………

Age……………………………………………………

Body height…………………………………………..

Body weight…………………………………………..

BMI (body mass index) ……………………………..

**Questionnaire**

1. What kind of work do you do?
   1. Physical
   2. Mental
   3. Mixed
2. What physical activity do you undertake regularly, i.e. min. 2 times a week? (more than one option can be selected):
   1. The gym
   2. Running
   3. Bicycle
   4. Fitness
   5. Other what? ……………………………………………………………
3. How do you rate your physical fitness?
   1. Very low
   2. Low
   3. Moderate
   4. High
   5. Very high

*Intense physical exertion causes very fast breathing and a very fast heartbeat - e.g. lifting heavy objects, digging the ground, aerobics, fast running, fast cycling. At least 10 minutes. continuously.*

1. How often do you engage in activities that require intense physical exertion?

a. ☐ Never / I'm not sure

b. ☐ Once a week or less

c. ☐ 2-3 times a week

d. ☐ 4 times a week or more

1. On average, how much time do you engage in intense physical exertion during such a day?

a. ………… .. minutes a day

b. ☐ I don't know / I'm not sure

*Moderate physical exertion leads to a little faster breathing and a little faster heartbeat - such as carrying lighter weights, cycling at a normal pace, playing volleyball or very brisk walking (not walking). At least 10 minutes. continuously.*

1. How often do you engage in activities that require moderate physical exertion?

a. ☐ Never / I'm not sure

b. ☐ Once a week or less

c. ☐ 2-3 times a week

d. ☐ 4 times a week or more

1. On average, how much time do you engage in moderate physical exertion during such a day?

a. ………… .. minutes a day

b. ☐ I don't know / I'm not sure

1. Physical activity, which at a time amounts to min. 45 minutes, heart rate increases, breathlessness develops, sweat on the body is felt:

a. ☐ Never / I'm not sure

b. ☐ Once a week or less

c. ☐ 2-3 times a week

d. ☐ 4 times a week or more

1. How often do you walk for at least 10 minutes. continuously? We are interested in work-related walking, walking down the street, e.g. shopping, going to work, and walking.

a. ☐ Never / I'm not sure

b. ☐ Once a week or less

c. ☐ 2-3 times a week

d. ☐ 4 times a week or more

1. How much time per day do you spend sitting, taking into account weekdays? We are interested in sitting at a desk, sitting while visiting friends, reading, and sitting or lying down while watching TV. Include time spent sitting at home, at work, at school, in vehicles, and elsewhere.

a. ☐ 4 hours and less

b. ☐ 5-6 hours

c. ☐ 7-8 hours

d. ☐ 9 hours and more

1. How often do you experience annoying overload and pain in the area of the spine, neck, head, shoulders (which go away on their own, but are unpleasant and last longer than a day)

a. ☐ Several times a week, almost every day

b. ☐ 1-2 times a week

c. ☐ Several times a month (3-4)

d. ☐ Very rarely, not every month

e. ☐ I don't experience it at all
